# Propionibacterium acnes overabundance and natural killer group 2 member D system activation in corpus‐dominant lymphocytic gastritis

**DOI:** 10.1002/path.4782

**Published:** 2016-10-21

**Authors:** Ana Montalban‐Arques, Philipp Wurm, Slave Trajanoski, Silvia Schauer, Sabine Kienesberger, Bettina Halwachs, Christoph Högenauer, Cord Langner, Gregor Gorkiewicz

**Affiliations:** ^1^ Institute of PathologyMedical University of GrazGrazAustria; ^2^ Theodor Escherich Laboratory for Medical Microbiome ResearchMedical University of GrazGrazAustria; ^3^ Centre for Medical ResearchMedical University of GrazGrazAustria; ^4^ Institute of Molecular BiosciencesUniversity of GrazGrazAustria; ^5^ BioTechMedInteruniversity CooperationGrazAustria; ^6^ Department of Internal Medicine, Division of Gastroenterology and HepatologyMedical University of GrazGrazAustria

**Keywords:** lymphocytic gastritis, 16S rRNA gene, stomach microbiota, Propionibacterium acnes, Helicobacter pylori, intraepithelial lymphocytes, NKG2D, MICA, IL‐15, short‐chain fatty acids, gastric epithelial cells

## Abstract

Corpus‐dominant lymphocytic gastritis (LyG) is characterized by CD8 ^+^ T‐cell infiltration of the stomach epithelium by a so far uncharacterized mechanism. Although Helicobacter pylori is typically undetectable in LyG, patients respond to H. pylori antibiotic eradication therapy, suggesting a non‐H. pylori microbial trigger for the disease. Comparative microbiota analysis of specimens from LyG, H. pylori gastritis and healthy controls precluded involvement of H. pylori in LyG but identified Propionibacterium acnes as a possible disease trigger. In addition, the natural killer group 2 member D (NKG2D) system and the proinflammatory cytokine interleukin (IL)‐15 are significantly upregulated in the gastric mucosa of LyG patients, and gastric epithelial cells respond to microbe‐derived stimuli, including live P. acnes and the microbial products short‐chain fatty acids, with induction of NKG2D ligands. In contrast, H. pylori infection does not activate or even repress NKG2D ligands. Together, our findings identify P. acnes as a possible causative agent for LyG, which is dependent on the NKG2D system and IL‐15 activation. © 2016 The Authors. *The Journal of Pathology* published by John Wiley & Sons Ltd on behalf of Pathological Society of Great Britain and Ireland.

## Introduction

Lymphocytic gastritis (LyG) accounts for up to 4.5% of chronic gastritis cases 1. Clinical symptoms range from abdominal pain and dyspepsia to severe cases with protein‐losing gastroenteropathy, weight loss, and anaemia 2. LyG is characterized histologically by an increased abundance of CD8^+^ intraepithelial lymphocytes (IELs), namely 25 per 100 epithelial cells (ECs) in the gastric epithelium (normal range <8 per 100 ECs). These IELs typically show a cytotoxic phenotype with granzyme B and T‐cell intracellular antigen‐1 expression 3, 4. Initially reported in the context of ‘varioliform gastritis’, LyG seems to be a histopathological syndrome rather than a single disease 1. Up to 45% of LyGs are associated with coeliac disease (CeD). In these cases, intraepithelial lymphocytosis is normally dominant in the gastric antrum. Several cases are thought to have been associated with *Helicobacter pylori* infection, although *H. pylori* is often not detectable 5. Rare causes include Crohn's disease, human immunodeficiency virus infection, common variable immunodeficiency, or the use of ticlopidine 2. Nevertheless, >20% of cases have an unknown aetiology, not associated with the above‐mentioned conditions. Interestingly, antibiotic therapy, namely *H. pylori* eradication therapy, seems to be an effective treatment for LyG, even in the absence of identifiable *H. pylori*, suggesting an alternative bacterial cause for the disease 6, 7, 8.

The molecular causes triggering the massive CD8^+^ IEL infiltration in LyG are also unknown. In CeD, the natural killer group 2 member D (NKG2D) system is critical for recruitment of CD8^+^ IELs and subsequent villus atrophy in the duodenum 9, 10. Natural killer (NK) cells, CD8^+^ T cells, γδ T cells, NKT cells and certain subsets of CD4^+^ T cells express the NKG2D receptor 11. The NKG2D receptor ligands (NKG2DLs) are expressed mainly on ECs at low levels under physiological conditions, but their expression is induced under conditions of cell stress, such as viral infection, neoplastic transformation, heat shock, or gliadin challenge 9, 10, 12, 13, 14. In humans, NKG2DLs include major histocompatibility complex (MHC) class I chain‐related protein A (MICA), MHC class I chain‐related protein B (MICB), and up to six different UL16‐binding proteins (ULBPs), also known as RAET1 proteins 11, 15. Upon ligand–receptor interaction, NKG2D triggers a cytotoxic response in the receptor‐bearing lymphocyte, eliminating the stressed cell that is overexpressing the ligand. This reaction is enhanced by the presence of the proinflammatory cytokine interleukin (IL)‐15 9, 16. Recently, it has been demonstrated that NKG2DL expression in the gastrointestinal (GI) mucosa is modulated by the gut microbiota 17. Moreover, short‐chain fatty acids (SCFAs) such as propionate and butyrate, which represent microbiota‐derived products, are potent inducers of NKG2DLs 18.

In the current study, we aimed to identify a possible bacterial trigger for LyG development by employing comparative microbiota analysis of stomach specimens obtained from persons with LyG, *H. pylori* gastritis (HpG), and healthy controls. Moreover, expression analysis of the NKG2D–NKG2DL system and the proinflammatory cytokine IL‐15 was used to assess activation of these molecular determinants that are needed for IEL infiltration. Finally, cell culture experiments were used to test whether gastric ECs are able to respond to microbial stimuli, including live *Propionibacterium acnes*, *H. pylori*, and the microbial products SCFAs, by induction of NKG2DLs.

## Materials and methods

### Ethics statement

The use of human tissue specimens was approved by the institutional review board of the Medical University of Graz (EK‐23‐212ex10/11).

### Specimens, histology, and immunohistochemistry

Formalin‐fixed paraffin‐embedded (FFPE) biopsy specimens were derived from the files of the Institute of Pathology of the Medical University of Graz (supplementary material, Table S1). Only cases with paired duodenal, gastric antral and gastric corpus specimens were included in the study. *H. pylori* carriage was determined by Warthin–Starry staining 19 and/or immunohistochemistry with an anti‐*H. pylori* antibody (clone SP48; Ventana, Tucson, AZ, USA). The following entities were used: healthy corpus (*n* = 24), corpus biopsies from corpus‐dominant LyG with proven absence of CeD (denoted LyG, *n* = 25), and corpus biopsies of H. pylori gastritis (denoted HpG, *n* = 25). Metadata and analyses performed on specimens are provided in supplementary material, Table S1. Sections from FFPE tissue specimens were stained with monoclonal mouse anti‐human CD8 (clone C8/144B; dilution 1:30; Dako Glostrup, Denmark), monoclonal mouse anti‐human CD4 (clone 4B12; dilution 1:20; Labvision, Fremont, CA, USA) and MICA/B (clone F‐6; dilution 1:200; Santa Cruz Biotechnology, Dallas, TX, USA) antibodies, according to the supplier's recommendations.

### Microbiota analysis

DNA extraction for microbiota analysis is described in Supplementary materials and methods. DNA quality and concentration were determined spectrophotometrically with a NanoDrop ND‐3300 instrument and the PicoGreen assay (Thermo Fisher Scientific, Waltham, MA, USA). Only specimens yielding an absorbance ratio of >0.8 at 260/280 nm and an absorbance ratio of ∼2 at 260/230 nm, respectively, were considered for further analyses. For amplification of the bacterial 16S rRNA gene FLX one‐way fusion primers (Lib‐L kit, Primer A, Primer B; Roche 454 Life Science, Branford, CT, USA) with the template‐specific sequence F27 and R357 (supplementary material, Table S2) targeting the V1–2 region of the 16S rRNA gene were used (amplicon length of 349 bp). Primers were chosen on the basis of their performance, enabling superior community capture and taxonomic resolution 20, and their good polymerase chain reaction (PCR) performance when applied to FFPE samples. PCR amplification was performed as described previously 21. Reactions for each sample were performed in triplicate, the quality of amplification products was checked visually on agarose gels, and only specimens resulting in reliable PCR amplification were used further. Amplicons were gel‐purified, pooled, and sequenced with the GS FLX Titanium Sequencing Kit XLR70 (Roche 454 Life Science), as described previously 21. For microbiota analysis, raw files from 454 FLX pyrosequencing were processed with MOTHUR v.1.31.2 according to the standard 454 SOP of MOTHUR 22. Sequencing errors were reduced with MOTHUR's implementation of PyroNoise 23, and the command pre.cluster 24 was used to remove sequences that arose because of pyrosequencing errors. Chimeras were removed with UCHIME 25, and non‐bacterial contaminants were removed by use of the Ribosomal Database Project (RDP) reference 26. The high‐quality reads were aligned to the SILVA database 27, 28. For operational taxonomic unit (OTU)‐based analyses, the processed fasta files from MOTHUR were introduced into QIIME v.1.7.0 29. OTUs were formed by clustering the sequences with uclust 30, with a similarity score of 97% (OTU 97% identity), and taxonomy was assigned by using the RDP classifier and Greengenes reference OTUs. A *de novo* OTU picking strategy was employed. The biomarker discover program LEfSe (linear discriminant analysis effect size) was used to determine differentially abundant OTUs 31. A batch file specifying the parameters used for microbiota analyses is given in Supplementary materials and methods. Differences in alpha‐diversity measures were tested by one‐way anova and a *post hoc* Bonferroni test. Principal coordinate analysis (PCoA) plots were created on the basis of a weighted‐unifrac 32 distance matrix, and statistical differences between groups were calculated with anosim. The presented values are always mean ± standard error of the mean if not indicated otherwise.

### Reverse transcription quantitative PCR (RT‐qPCR)

Total RNA from FFPE samples (10 sections, each 5 µm in thickness) was isolated with deparaffinization solution (Qiagen, Hilden, Germany) and the RNeasy FFPE kit, which includes a DNase treatment step (Qiagen). RNA from cell culture experiments was extracted by the use of TRIzol (Thermo Fisher Scientific) and the PureLink RNA mini kit (Invitrogen), according to the manufacturer's specifications. RNA quality and quantity were determined spectrophotometrically by the use of a NanoDrop instrument (ThermoScientific), as described above, and 1 µg of total RNA was used for cDNA synthesis with the GeneAmp RNA PCR kit (Thermo Fisher Scientific), according to the manufacturer's instructions. Quantitative real‐time PCR was performed with an ABI PRISM 7900HT instrument (Applied Biosystems) and SYBR Green PCR core reagents (Applied Biosystems). The oligonucleotide primers used are shown in supplementary material, Table S2. Reaction mixtures were incubated for 10 min at 95 °C, and this was followed by 40 cycles of 15 s at 95 °C, 1 min at 60 °C, and finally 15 s at 95 °C, 15 s at 60 °C, and 15 s at 95 °C. For each mRNA target, the expression level was normalized by using the β‐actin gene (*ACTB*) as a reference, and ratios were calculated with Pfaffl's method 33. For determination of *P. acnes* loads, 50 ng of total DNA extracted from the FFPE specimens was used as a normalized input for real‐time PCR amplification. Each PCR reaction was performed in triplicate, and analyses were repeated three times.

### Bacterial culture

*P. acnes* strains originating from the human stomach were kindly provided by B Mayo 34, and cultured under anaerobic conditions (Genbox anaer; bioMerieux, Marcy l'Etoile, France) on Columbia blood agar plates (bioMerieux) at 37 °C. *H. pylori* strains PMSS1 35 and SS1 36 were routinely grown on Columbia blood agar plates at 37 °C for 3 days in a microaerobic atmosphere (Genbox microaer; bioMerieux) prior to gastric cell line infection. *Escherichia coli* DSM30083 (purchased from DSMZ, Braunschweig, Germany) and *E. coli* DH5α 37 were cultured routinely on Columbia agar plates (bioMerieux) under aerobic conditions at 37 °C.

### Cell culture, infection, and SCFA stimulation assays

AGS cells were obtained from Cell Lines Service (Eppelheim, Germany). MKN28 cells were originally obtained from the Japanese Collection of Research Bioresources (JCRB; http://cellbank.nibio.go.jp/) and were kindly provided by S Wessler 38. Epithelial cells were seeded at 1.5 × 10^5^ per well of six‐well plates in 3 ml of Dulbecco's modified Eagle's medium (DMEM) high glucose (4.5 g/l) (GE Healthcare, Vienna, Austria), 10% fetal bovine serum (FBS) (Thermo Fisher Scientific) and 5 mm l‐glutamine (PAA, Vienna, Austria), and were grown to 80% confluence in a water‐saturated atmosphere of 95% air and 5% CO_2_ at 37 °C. Prior to the infection assays, a single *E. coli* colony was transferred into a 15‐ml tube containing 3 ml of Brucella broth (Roth, Karlsruhe, Germany), and incubated with gentle agitation (100 r.p.m.) at 37 °C for 4 h. For *P. acnes* infection, a bacterial suspension with an OD_600 nm_ of 0.1 [corresponding to 10^8^ colony‐forming units (CFUs)/ml] was cultivated for 24 h in wells of six‐well plates containing 3 ml of DMEM high glucose (4.5 g/l), containing 10% FBS and 5 mm l‐glutamine. Subsequently, AGS and MKN28 cells were infected with *P. acnes*, *H. pylori* or *E. coli* at a multiplicity of infection (MOI) of 1:50 for 24 h. Measurement of SCFAs in co‐culture supernatants by gas chromatography–mass spectrometry is described in Supplementary materials and methods. For SCFA stimulation, cells (1.2 × 10^6^/well) were incubated with 5 mm propionate, butyrate, acetate, or hydrochloric acid (Sigma Aldrich, St. Louis, MO, USA) for 4 h 39, 40, 41. Subsequently, cells were harvested by gentle centrifugation 300 g, 2 min and stored in 500 µl of Trizol (Thermo Fisher Scientific) for RNA isolation. Cells used for protein expression were rescued after 4 h of stimulation with SCFAs in DMEM high glucose (10% FBS, 5 mm l‐glutamine) for another 4 h before being analysed by flow cytometry 18. Experiments were performed in triplicate and repeated three times.

### Flow cytometry

AGS and MKN28 cells were harvested in ice‐cold phosphate‐buffered saline, because trypsin cleaves surface NKG2DLs, giving false‐negative results 42. A detailed protocol specifying the preparation steps for flow cytometry is given in Supplementary materials and methods.

### Statistical analysis

Quantitative PCR and flow cytometry data were assessed with the D'Agostino & Pearson test for their normal distribution. Data are given as mean ± standard deviation if not otherwise specified. Statistical analyses were performed with GraphPad Prism 5 software, by the use of one‐way anova and either Tukey's *post hoc* test (for FFPE samples) or Dunnett's *post hoc* test (for *in vitro* and flow cytometry assays). *p*‐Values of <0.05 were considered to be statistically significant.

### Data deposition

The sequencing data generated for this work can be accessed via the EBI short read archive (EBI SRA) under the accession number ERP013255.

## Results

### LyG is not associated with H. pylori infection but is signified by P. acnes overabundance

**Figure 1:**
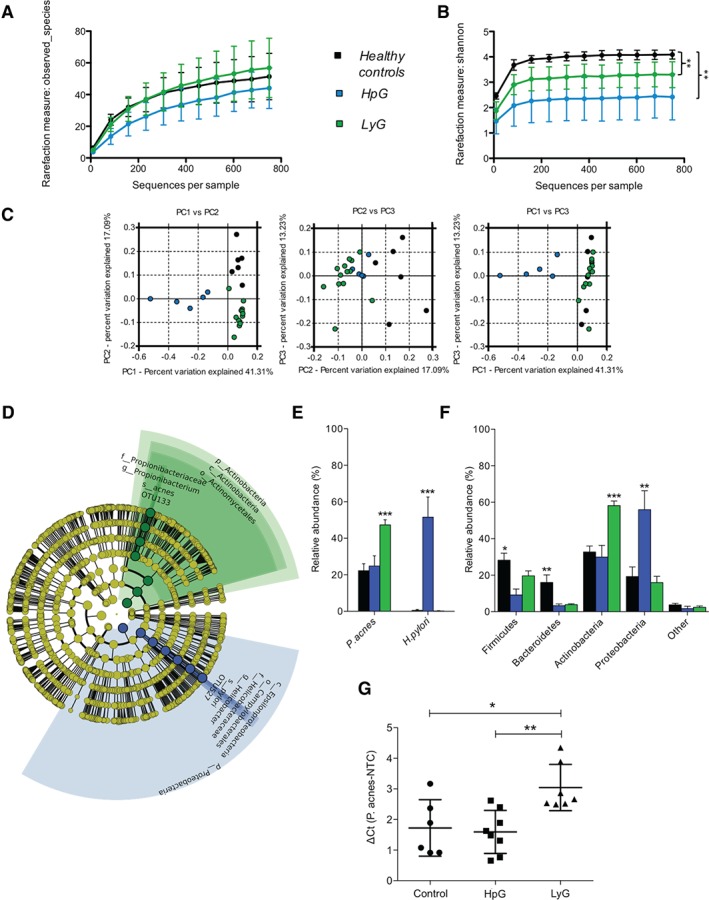
Comparative microbiota analyses of LyG, HpG and healthy controls. (A) Microbial richness (i.e. number of identifiable taxa) showed no statistically significant difference between entities. (B) The Shannon diversity index was significantly lower in HpG and LyG than in controls. (C) Significantly different microbial community structures in HpG, LyG and healthy controls, as indicated by distinct clustering in PCoA (measure: weighted unifrac; anosim, p < 0.001). (D) Cladogram representing the LEfSe output comparing controls, HpG and LyG. Only two highly significant associations with entities (LDA score of >5.1) were found, namely H. pylori in HpG and P. acnes in LyG. The diameter of each circle is proportional to the taxon's abundance in the graph. (E) Relative abundance of P. acnes and H. pylori in healthy controls, HpG, and LyG. (F) Relative abundance of the main bacterial phyla in healthy controls, HpG samples, and LyG samples (*p < 0.05, **p < 0.01, ***p < 0.001; according to LEfSe analysis encompassing the Kruskal–Wallis and Wilcoxson tests). (G) Quantitative PCR results validating P. acnes overabundance in LyG (*p < 0.05; **p < 0.01; one‐way anova and Tukey's post hoc test).

To investigate the gastric microbiota in LyG and to discern a possible bacterial disease trigger, we subjected gastric corpus biopsies originating from LyG (*n* = 13), HpG (*n* = 5) and healthy controls (*n* = 6) to comparative 16S rRNA gene profiling; 4841 ± 2309 reads were generated per sample, corresponding, on average, to 74 ± 28 OTUs (97% identity) per sample. Microbial richness, which is a measure of how many taxa are detectable in the respective sample, showed no difference between entities (Figure 1A). In contrast, diversity and evenness, which are measures of how diverse a microbial community is and how equally the taxa therein are distributed, were significantly lower in HpG and LyG than in controls (Figure 1B; supplementary material, Table S3). This finding suggests that, in LyG, similarly to HpG, certain bacteria may dominate the microbial community. PCoA (measure: weighted unifrac distance) indicated significantly different microbial community structures (anosim, *p* < 0.001) of entities (Figure 1C). Comparative analysis with LEfSe revealed certain significantly different abundant phylotypes in entities (supplementary material, Table S4). Importantly, only two OTUs showed a markedly high linear discriminant analysis (LDA) score (LDA of >5.1) and statistical significance. *H. pylori* OTU527 showed significantly increased abundance in HpG (*p* = 0.0003), and *P. acnes* OTU133 showed significantly increased abundance in LyG (*p* < 0.0006; Figure 1D). *P. acnes* accounted for 47.36 ± 2.74% of taxa in LyG, 22.23 ± 3.82% in controls, and 24.77 ± 5.63% in HpG (Figure 1E). *H. pylori* accounted for 51.54 ± 11.11% of taxa in HpG, but was nearly absent in LyG and controls. Only one healthy control (specimen 13) and two LyG specimens (specimens 32 and 43) contained *H. pylori* at low abundance (0.49 ± 1.2% and 0.18 ± 0.62%, respectively). Low‐level colonization of asymptomatic individuals with *H. pylori* has been described recently 43, 44, 45, 46. The taxonomic differences were also reflected at the phylum level, wherein HpG showed a significant relative increase in the abundance of proteobacteria (55.91 ± 8.45%); 89.95 ± 7.22% of proteobacterial reads originated from *H. pylori*. LyG showed a significant relative increase in the abundance of actinobacteria (58.12 ± 2.56%); 81.36 ± 8.68% of them were represented by *P. acnes*. Accordingly, the *Firmicutes* and *Bacteroidetes* were significantly depleted in HpG and LyG as compared with controls (Figure 1F; supplementary material, Figure S1). Finally, quantitative PCR performed on LyG, HpG and controls confirmed significantly increased *P. acnes* loads in LyG (Figure 1G). Moreover, a significant correlation of abundance determined by 16S next‐generation sequencing and load determined by quantitative PCR was evident, validating the microbiota analysis results (supplementary material, Figure S2). Collectively, these data indicate that LyG is not associated with *H. pylori* infection, but shows significantly increased *P. acnes* loads.

### The NKG2D–NKG2DL system is induced in LyG but not in HpG

**Figure 2:**
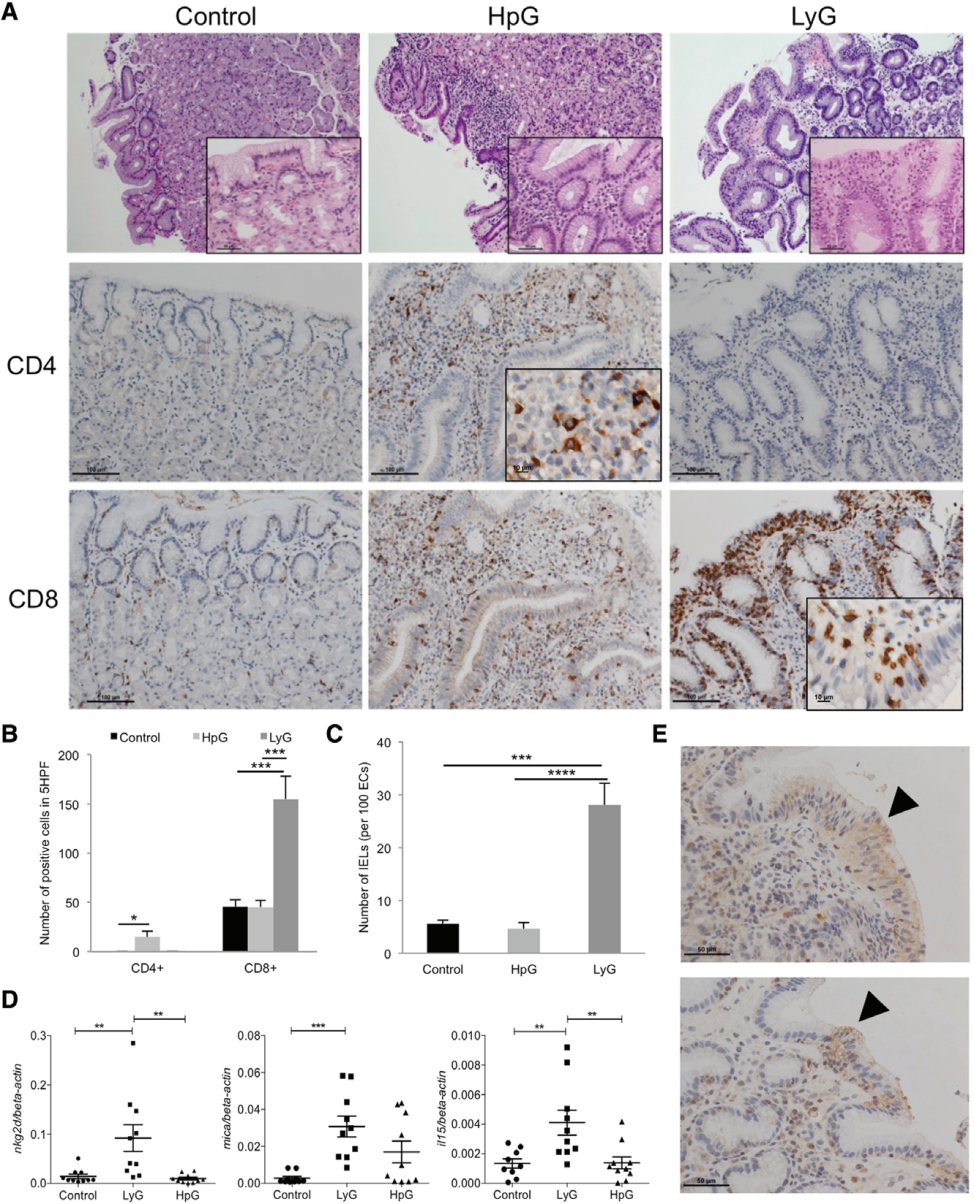
Immunophenotype and gene expression of the NKG2D–NKG2DL system and IL‐15 in gastric corpus biopsies. (A) Haematoxylin and eosin and immunohistochemical staining of CD4^+^ and CD8^+^ T cells in human corpus biopsies of healthy controls, HpG cases, and LyG cases (overall magnification: upper panels, ×100; insets, ×400; middle and lower panels, ×200; immunohistochemistry insets, ×600). (B) IELs in LyG are CD8^+^ and significantly enriched as compared with HpG and healthy controls. In HpG, CD4^+^ cell numbers in the lamina propria are significantly increased as compared with LyG and healthy controls. (C) Number of IELs in healthy controls, HpG and LyG cases. (D) mRNA expression of NKG2D, MICA and IL‐15 indicates significant upregulation in LyG as compared with HpG and controls (n = 10 each). (E) Enhanced staining with a MICA/B antibody in gastric corpus biopsies of LyG cases in areas wherein IELs are more abundant (arrow) (magnification: ×400; *p < 0.05, **p < 0.01, ***p < 0.001, ****p < 0.0001; one‐way anova and Tukey's post hoc test).

The NKG2D–NKG2DL system and the proinflammatory cytokine IL‐15 are major determinants for IEL recruitment in the gut. Upregulation of both factors leads to intraepithelial lymphocytosis, and subsequently to villus atrophy, in CeD 9, 10. The phenotypic similarities between LyG and CeD prompted us to investigate the involvement of this cell stress‐sensing system in LyG. First, we comparatively assessed the number of CD8^+^ and CD4^+^ lymphocytes in gastric corpus specimens from LyG, HpG and healthy controls by immunohistochemistry (Figure 2A). LyG cases showed significant increases in the numbers of CD8^+^ lymphocytes as compared with HpG and healthy controls (Figure 2B). These CD8^+^ T cells were mainly IELs. The average CD8^+^ IEL counts were 28.04 ± 4.15 per 100 ECs in LyG, 5.6 ± 0.62 per 100 ECs in healthy controls, and 4.64 ± 1.18 per 100 ECs in HpG (Figure 2C). In contrast, HpG showed a significant increase in the number of CD4^+^ T cells [14.4 ± 5.65 in five high‐power fields (HPFs)] as compared with healthy controls; the CD4^+^ T cells were mainly present in the lamina propria (Figure 2A, B). Next, we comparatively assessed the expression of *NKG2D*, NKG2DLs (*MICA*, *MICB*, *ULBP1*, *ULBP2*, *ULBP3* and *ULBP4*) and *IL‐15* by RT‐qPCR. Gastric corpus biopsies of LyG showed significant overexpression of *NKG2D* and *IL‐15* mRNA as compared with HpG and healthy controls, and *MICA* levels were significantly increased as compared with healthy controls (Figure 2D). *MICB*, *ULBP1* and *ULBP2* mRNA expression was slightly repressed in LyG (supplementary material, Figure S3). It is of note that HpG showed no significant induction of the expression of assessed markers, which correlated with the observed absence of CD8^+^ T‐cell infiltration in HpG (Figure 2B). Gastric corpus biopsies of LyG also showed pronounced staining with an MICA/B antibody in areas wherein the numbers of IELs were increased, indicating induction of the system in the epithelium (Figure 2E). Taken together, these data indicate that expression of the NKG2D–NKG2DL system and of the proinflammatory cytokine IL‐15 are induced in LyG, suggesting that these factors are important for CD8^+^ IEL recruitment and disease pathogenesis. Interestingly, NKG2D–NKG2DL system and IL‐15 expression are not induced in HpG, pointing towards deviating mucosal immune reactions and pathogeneses of both diseases.

### Gastric epithelial cells respond to challenge with P. acnes and SCFAs by induction of NKG2D ligand expression, whereas H. pylori does not induce ligand expression

**Figure 3:**
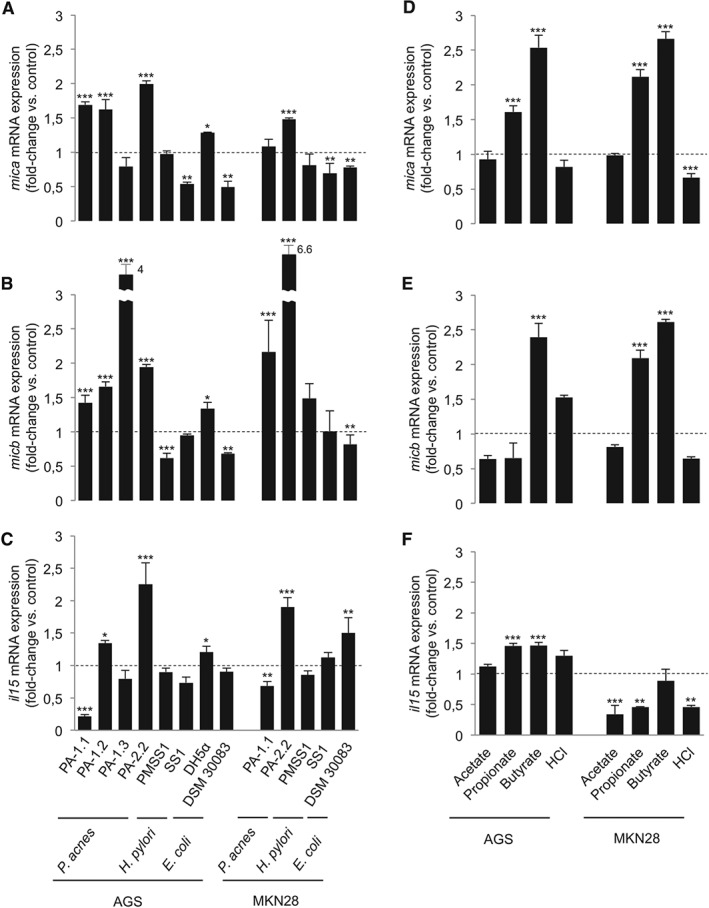
Gastric epithelial cells respond to P. acnes and SCFAs by induction of NKG2DL and IL‐15 mRNA expression, whereas H. pylori does not cause such induction. (A–C) AGS and MKN28 cells were infected with live bacteria for 24 h. MICA (A), MICB (B) and IL‐15 (C) mRNA relative abundance was measured by RT‐qPCR. P. acnes strains isolated from the human stomach are able to induce NKG2DL expression in a strain‐dependent manner, and had either inducing or repressing effects on IL‐15 expression. H. pylori challenge does not induce NKG2DL mRNA expresion or even represses transcription. (D–F) AGS and MKN28 cells were stimulated with 5 mm of different SCFAs and HCl (control). MICA (D), MICB (E) and IL‐15 (F) mRNA expression was assessed after 4 h of challenge. Propionate and butyrate induced NKG2DL expression in both cell lines. SCFAs had either inducing or repressing effects on IL‐15 mRNA transcript abundance, depending on the cell line (*p < 0.05; **p < 0.01; ***p < 0.001; one‐way anova and Dunnett's post hoc test).

It has been shown that microbes are able to induce NKG2DL expression in certain cell lines; however, human gastric epithelia have not been investigated for their responsiveness thus far 17, 18. Therefore, we challenged AGS and MKN28 cells with *P. acnes* strains isolated from the human stomach, both *H. pylori* and *E. coli*, for 24 h (MOI of 1:50). After 24 h of challenge, *MICA*, *MICB* and *IL‐15* expression was measured by RT‐qPCR. Shorter co‐culture times (4 h) did not substantially alter expression of the evaluated markers (supplementary material, Figure S4A). The growth of assessed strains determined by CFU plating was not significantly different after 24 h of co‐cultures (supplementary material, Figure S4B). Live *P. acnes* significantly increased *MICA* and *MICB* mRNA levels in both gastric epithelial cell lines in a strain‐dependent manner. For instance, strain PA2‐2 consistently showed strong induction of ligand expression and also significantly induced *IL‐15* mRNA expression, whereas PA1‐1 significantly repressed *IL‐15* expression. It is noteworthy that *H. pylori* strains SS1 and PMSS1 did not induce, but rather repressed, ligand and *IL‐15* mRNA expression in both cell lines. The effect of *E. coli* challenge on mRNA levels was only minor as compared with *P. acnes* (Figure 3A–C).

It has been recently reported that SCFAs, including propionate derived from *P. acnes*, are potent inducers of NKG2DL expression 18. SCFAs could be reliably detected in supernatants after a 24‐h AGS challenge with *P. acnes* (supplementary material, Table S5). However, their concentration was approximately 1 to 2 log units lower than the concentration normally needed to reliably induce NKG2DL expression *in vitro* 18, 47, 48. Thus, it is reasonable to speculate that other factors, such as direct bacterium–cell contact or other metabolites, also contributed to the observed induction of ligand expression in our challenge experiments 49. To assess the net effect of propionate, and also the effect of the potent NKG2DL inducer butyrate, AGS and MKN28 cells were challenged with 5 mm SCFAs and HCl as a control for 4 h, and *MICA*, *MICB* and *IL‐15* expression was assessed by RT‐qPCR. Butyrate and propionate significantly induced *MICA* expression in both gastric epithelial cell lines and *MICB* expression in MKN28 cells. Both SCFAs also induced *IL‐15* mRNA expression in AGS cells. MKN28 cells responded differently, showing no effect on *IL‐15* mRNA expression or even repression. Neither acetate nor HCl changed the expression of ligands and *IL‐15* mRNA in AGS cells, but repressed *IL‐15* mRNA expression in MKN28 cells (Figure 3D–F).

**Figure 4:**
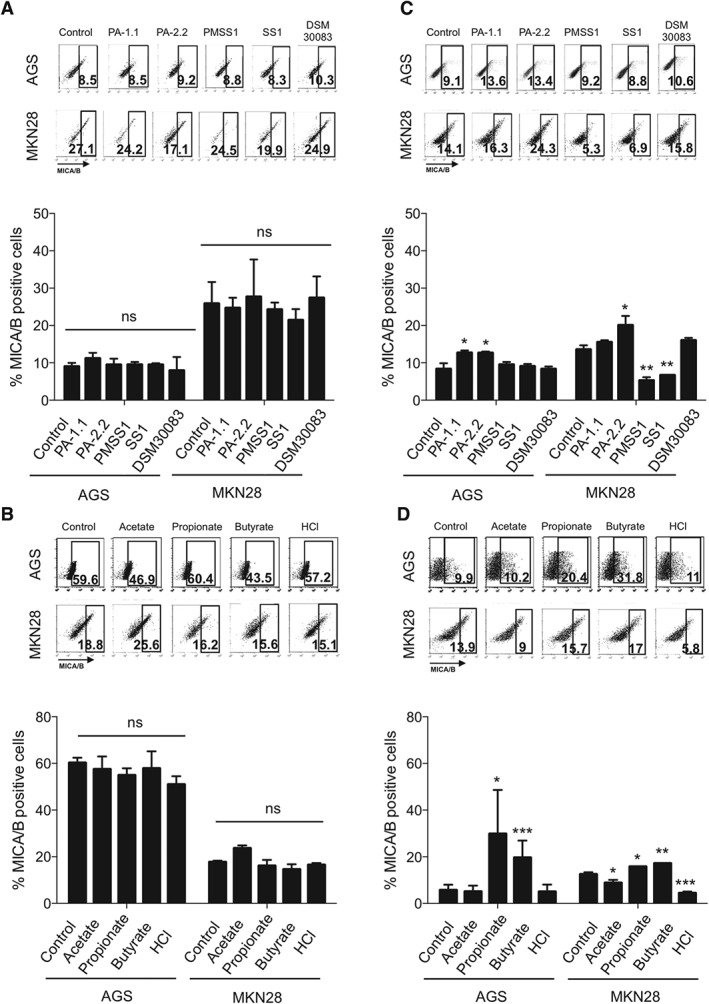
Gastric epithelial cells respond to P. acnes and SCFAs by induction of NKG2DL expression specifically on the cell surface, whereas H. pylori does not cause such induction. MICA/B protein expression was assessed in AGS and MKN28 cells by flow cytometry after challenge with bacteria for 24 h or SCFA stimulation for 4 h followed by 4 h of rescue in DMEM without SCFAs. Representative pictures of dot plots are shown at the top of each graph. (A) No change in overall MICA/B protein expression after bacterial challenge with P. acnes, H. pylori and E. coli strains. (B) No change in overall MICA/B protein expression after challenge with 5mM SCFAs and HCl (C) Significant induction of extracellular MICA/B protein expression after P. acnes challenge in AGS and MKN28 cells. H. pylori suppresses extracellular MICA/B expression in MKN28 cells. (D) Propionate and butyrate induce extracellular MICA/B protein; 10 000 events were analysed per readout. Readouts of concomitantly performed viability assays of AGS and MKN cells are shown in supplementary material, Figure S5. Bar charts represent the mean values of percentages of MICA/B‐positive cells ± standard error of three independent experiments (*p < 0.05; **p < 0.001; ***p < 0.0001; one‐way anova and Dunnett's post hoc test). NS, not significant.

It is of note that NKG2DL expression is differently regulated at the mRNA and protein levels 50. Thus, we wanted to investigate whether challenge also translates into increased protein expression of NKG2DLs, which would be necessary for recruitment of NKG2D receptor‐bearing lymphocytes to the ligand‐overexpressing epithelium. By using flow cytometry and a MICA/B antibody, we found that challenge of AGS and MKN28 cells with live bacteria for 24 h (Figure 4A) or with 5 mm acetate, propionate, butyrate or HCl for 4 h (Figure 4B) did not alter overall ligand expression.

However, challenge experiments did show effects on extracellular ligand expression. *P. acnes* strains significantly induced extracellular MICA/B expression in both cell lines, whereas *H. pylori* strains did not alter (for AGS) or even repressed (for MKN28) extracellular MICA/B protein expression (Figure 4C). In addition, propionate and butyrate significantly increased extracellular MICA/B protein levels in both cell lines, whereas acetate and HCl did not alter (for AGS) or slightly repressed (for MKN28) extracellular MICA/B protein levels (Figure 4D). Taken together, these data indicate that live *P. acnes*, its main metabolite propionate and the SCFA butyrate are potent inducers ofNKG2DLs mRNA and extracellular protein expression, and also modulate *IL‐15* mRNA levels in human gastric epithelial cells. Intriguingly, *H. pylori* does not induce, or even represses, mRNA and protein expression of NKG2DLs and IL‐15.

## Discussion

The pathogenesis of corpus‐dominant LyG is so far unknown, but its responsiveness to antibiotic treatment suggests a bacterial trigger for disease development 6, 7, 8. In this study, we subjected human stomach biopsies to comparative microbial community profiling, and found that *H. pylori* infection is not the cause of LyG, which is instead characterized by overabundance of *P. acnes*. Moreover, we found expression of the NKG2D–NKG2DL system and the proinflammatory cytokine IL‐15 to be significantly induced in LyG, identifying the likely molecular determinants responsible for IEL recruitment, the typical phenotype represented by the disease. Finally, challenge of human gastric ECs with *P. acnes* and the microbial metabolites SCFAs revealed induction of NKG2DL expression, recapitulating the measurements found in human disease specimens. It is of note that *H. pylori* did not induce, or even repressed, NKG2DL expression.

Immune recognition mediated by the activating receptor NKG2D plays an important role in the elimination of stressed cells. NKG2DLs are expressed at low levels on epithelia under healthy conditions; however, their expression is greatly enhanced by factors causing cell stress, such as viral infection, heat shock, or neoplastic transformation 50, 51. In CeD, duodenal epithelia challenged with gliadin (i.e. the stressor) overexpress the NKG2DL MICA. In the presence of IL‐15, cytotoxic CD8^+^ lymphocytes expressing the activating receptor NKG2D are then recruited to the duodenal epithelium, leading to the destruction of stressed cells via a cytotoxic T‐cell response, which subsequently leads to villus atrophy, the hallmark lesion observed in CeD 9, 10. Recently, it has been shown that NKG2DL expression is modulated by the GI microbiota, either by direct microbe–cell interaction (e.g. via adherent *E. coli*) or by microbial products such as SCFAs 18, 49. Moreover, manipulation of the microbiota with antibiotics leads to either increased or decreased NKG2DL expression in mice, depending on the microbial spectra covered by the substance 17.

Historically, it has been considered that the stomach is a quasi‐sterile environment, owing to its acidity, and that only bacteria with specific abilities (e.g. *H. pylori* with its urease production) are able to colonize this habitat. However, it has now become clear that the stomach's microbiota is quite diverse, and that it also contributes to the development of various gastric pathologies 44, 45, 52, 53. It is of note that *P. acnes*, a classic skin bacterium, has been recently identified as a part of the stomach microbiota 43, 52, 54, 55. By the use of culture‐dependent and culture‐independent techniques, *P. acnes* was found to be a member of the stomach microbiota in healthy individuals, representing >20% of microbes in certain cases 55. Interestingly, *P. acnes* was found only in mucosal specimens and not in the gastric fluid, indicating the preferred niche of this bacterium 56. Whether specific pathotypes of *P. acnes* contribute to LyG development, or whether the increase in the abundance of *P. acnes* over a certain level is itself sufficient to induce NKG2DL overexpression, is not known so far, and should be a focus for future investigations. Nevertheless, *P. acnes* is able to resist acid stress, and it shows a variety of virulence mechanisms, which could contribute to inflammation, epithelial cell stress, and ultimately to NKG2D–NKG2DL activation 34, 57, 58, 59, 60, 61, 62. Interestingly, in HpG (a condition certainly favouring cell stress of gastric epithelia, owing to its prominent inflammation), neither NKG2D nor MICA or IL‐15 expression were found to be induced. In contrast, we noted only slight induction of MICB expression in biopsies. Moreover, *H. pylori* failed to induce or even impaired mRNA and extracellular protein expression in challenge experiments with gastric epithelial cells. Importantly, the NKG2D–NKG2DL system and IL‐15 are important for tumour surveillance, which is necessary for the elimination of neoplastic cells 63. The system has therefore been investigated as a potent target for cancer immunotherapy in various studies 64, 65, 66, 67, 68. From our data, it could be speculated that *H. pylori* does not have the ability to activate the NKG2D–NKG2DL system, and this might eventually favour stomach cancer development as a long‐term sequel of *H. pylori* infection, because of impaired innate antitumour immunity. The downregulation of *IL‐15* in HpG has also been reported recently 69. Thus investigating the NKG2D–NKG2DL system in the context of HpG and gastric adenocarcinoma development should be a reasonable future research aim.

In conclusion, our study identifies the NKG2D–NKG2DL system and the proinflammatory cytokine IL‐15 as likely molecular players in corpus‐dominant LyG. Thus, similarities between LyG and the paradigm disease of intraepithelial lymphocytosis, CeD, also exist at the molecular level. The identified increase in *P. acnes* abundance in LyG possibly contributes to pathogenesis, as also shown by the *in vitro* cell challenge experiments. Identifying the causes leading to *P. acnes* overgrowth or which additional factors contribute to NKG2D–NKG2DL and IL‐15 activation should initiate prospective studies investigating LyG. This would enable, for instance, genotyping and phenotyping of *P. acnes* isolates from cases, which is not feasible with archived FFPE material.

## Author contributions statement

The authors contributed in the following way: GG, CL, AM‐A: conceptualization and methodology; AM‐A, PW, ST, SS, GG: investigation and formal analysis; GG, AM‐A: writing of original draft; AM‐A, PW, ST, BH, SK, CH, GG: writing, review and editing; GG: funding acquisition; GG, CH, BH: resources; GG: supervision.

**SUPPLEMENTARY MATERIAL ONLINE**

**Supplementary materials and methods**

**Supplementary figure legends**

**Figure S1.** Differences at phylum level between healthy controls, HpG and LyG.

**Figure S2.** Validation of NGS sequencing results by qPCR.

**Figure S3.** NKG2DL expression in corpus biopsies measured by qRT‐PCR.

**Figure S4.** AGS cell challenge for 4 h and bacterial viable cell counts.

**Figure S5.** Apoptosis and live/dead staining assay.

**Table S1.** Sample information, metadata and analyses performed.

**Table S2.** Primers used in this study.

**Table S3.** Richness, diversity, evenness.

**Table S4.** LEfSe analysis output.

**Table S5.** Concentration of SCFAs in the supernatant of challenged AGS cells.

## Supporting information


**Supplementary materials and methods**

**Supplementary figure legends**

**Figure S1. Differences at phylum level between healthy controls, HpG and LyG.** Relative abundance of Actinobacteria is significantly increased in LyG compared to healthy controls and HpG. Relative abundance of Proteobacteria is significantly increased in HpG compared to healthy controls and LyG samples. Relative abundance of Bacteroidetes is significantly decreased in HpG and LyG. Relative abundance of Firmicutes is significantly decreased in HpG. Data represent the mean ± SEM. **p<0.01, ***p<0.001, ****p<0.0001 by one‐way ANOVA and post‐hoc Bonferroni's test.

**Figure S2. Validation of NGS sequencing results by qPCR.** Spearman correlation analysis (non‐parametric) of samples with paired 16S rRNA gene sequencing and qPCR data shows a significant correlation of relative abundance and load (Ct value).

**Figure S3. NKG2DL expression in corpus biopsies measured by qRT‐PCR.** micb mRNA is significantly increased in HpG samples compared to LyG. Among the ULBPs, ulbp1 and ulbp4 are down‐regulated in LyG compared to control, while HpG samples show similar expression levels as healthy controls. (n=10). Data represent the mean ± SEM. *p<0.05, by One‐way ANOVA and post‐hoc Tukey's test.

**Figure S4. AGS cell challenge for 4 h and bacterial viable cell counts. (A)** mica, micb and il15 mRNA gene expression in AGS cells after 4h of infection with P. acnes and E. coli strains **(B)** Colony‐forming‐units per ml (CFU/ml) of P. acnes, H. pylori and E. coli (DSM 30083) strains after 24 h of co‐cultivation with AGS (left) or MKN28 (right) cells. Bars show the mean ±SD, by One‐way ANOVA and post‐hoc Tukey's test. ns: not significant

**Figure S5. Apoptosis and live/dead staining assay. (A)** AGS and **(B)** MKN28 cells were infected with different bacteria (E. coli denotes DSM30083) for 24h and assessed by Annexin V/PI staining and flow cytometry. **(C)** AGS and **(D)** MKN28 cells were stimulated with 5 mM of different SCFAs or HCl for 4h and assessed by Annexin V/PI staining and flow cytometry. Bar charts represent three independent Annexin V/PI experiments showing the percentage of viable (Annexin V‐/PI‐), apoptotic (Annexin V+/PI‐) and dead (Annexin V+/PI+) cells, respectively. Bars show the mean ±SD. *p<0.05, ** p<0.001,***p<0.000, by one‐way ANOVA and post‐hoc Dunnett's test.

**Table S1** Sample information, metadata and analyses performed.

**Table S2.** Primers used in this study

**Table S3.** Richness, diversity, evenness

**Table S4.** LEfSe analysis output.

**Table S5.** Concentration of SCFAs in the supernatant of challenged AGS cells assessed by GC‐MS.
