# Supplementary material for: Propionibacterium acnes overabundance and natural killer group 2 member D system activation in corpus‐dominant lymphocytic gastritis
Source: J Pathol. 2016 Oct 21;240(4):425–36. doi: 10.1002/path.4782 (PMC5111592; doi:10.1002/path.4782)
Supplement: Supplementary file 1 — Supplementary materials and methods [file PATH-240-425-s009.docx]

**Supplementary materials and methods**

**DNA extraction for microbiota analysis.** For DNA extraction, FFPE blocks were cut into 5 µm thick sections. The first 5 sections were discarded and the subsequent 10 sections were sampled, incubated with 400 µl xylene and centrifuged at 13,000 rpm in a table-top centrifuge at room temperature (RT) for 10 min. Xylene was discarded and this step was repeated 3 times. Thereafter, 400 µl absolute ethanol were added, the mixture gently vortexed and centrifuged at 13,000 rpm in a table-top centrifuge at RT for 10 min. This step was repeated 2 times and the pellet was air-dried for 2 h. Subsequently, samples were subjected to mechanical lysis with a MagNA Lyser Instrument (Roche Diagnostics, Mannheim, Germany) and total DNA was isolated with the MagNA Pure LC DNA Isolation Kit III (bacteria, fungi) in a MagNA Pure LC 2.0 Instrument (Roche Diagnostics). Therefore, tissue pellets were mixed with 230 µl bacterial lysis-buffer and transferred to MagnaLyser tubes and centrifuged at 6500 rpm for 20 s. Thereafter, 5.75 µl lysozyme (100 mg/ml) were added and the mixture was incubated at 37°C for 30 min. Subsequently, 20 µl proteinase K were added according to the MagNA Pure protocol and the mixture was incubated at RT overnight. Then, samples were incubated at 95°C for 10 min, spun down and placed on ice for 5 min. Finally, samples were centrifuged at 13,000 rpm in a table-top centrifuge and 100 µl of the lysates were transferred into MagnaPure sample tubes and further processed according to the manufacturer’s instructions.

**Short-chain fatty acid measurements by GC-MS.** Acetate, propionate and butyrate levels from culture supernatants were measured by GC-MS using a Thermo Scientific DSQ II™ Series Single Quadrupole GC-MS by electron ionization instrument. Supernatant samples (500 µl) were mixed with 500 µl H_3_PO_4_ (0.5%), internal standards (ISTD: 200 µl dC2 [500 µM] and 200 µl dC4 [500 µM]) and 600 µl methyl tert-butyl ether (MTBE). After centrifugation (10 min; 2.500 rpm; RT) 150 µl of the organic phase were used for the measurement. A calibration curve was created with increasing ISTDs mixed with H_3_PO_4_ (0,5%) for the analysis.

**Flow cytometry.** For detection of membrane-bound MICA/B protein, cells were incubated with 5 µl (0.125 µg) of the anti-human monoclonal MICA/B Alexa-Fluor® 647 antibody (clone 6D4, mouse IgG2a, kappa; BioLegend, CA, USA) diluted in 45 µl PBS at 4ºC for 30 min. Subsequently, cells were washed twice in 500 µl PBS at RT. For detection of intracellular MICA/B protein, cells were fixed by adding 500 µl of IC fixation buffer (eBioscience, CA, USA) and incubated in the dark at RT for 30 min. Afterwards, cells were permeabilized using 1X permeabilization buffer (eBioscience) and stained using 5 µl (0.125 µg) of antibody diluted in 1X permeabilization buffer in a final volume of 50 µl. Thereafter, cells were incubated in the dark at RT for 30 min. Then, cells were washed twice in 1X permeabilization buffer and resuspended in PBS for analyses. Additionally, 7-AAD viability staining solution (eBioscience) or the Annexin V Apoptosis Detection Kit APC (eBioscience) were used following the manufacturer’s recommendations to check cell viability and apoptosis. Cells were analysed with a FACS bench-top cytometer (BD™ LSRII) according to the manufacturer’s recommendations and the results were calculated using CELLQuest (BD Bioscience) and FlowJo (Tree Star, Ashland, OR, USA).

**Microbiota Analysis**

Batch file specifying parameters used for microbiota analyses. Parameters other than default settings are shown in parentheses

MOTHUR (v.1.31.2)

sffinfo(flow=T)

trim.flows(pdiffs=2, bdiffs=1, minflows=360, maxflows=720)

shhh.flows()

trim.seqs(pdiffs=2, bdiffs=1, maxhomop=8, minlength=200)

unique.seqs()

align.seqs(reference=silva.bacteria.fasta)

screen.seqs(start=1044, optimize=end, criteria=95)

filter.seqs(vertical=T, trump=.)

unique.seqs()

pre.cluster()

chimera.uchime()

remove.seqs(dups=T)

classify.seqs(template=trainset9_032012.pds.fasta, taxonomy=trainset9_032012.pds.tax, cutoff=80)

remove.lineage(taxon=Mitochondria-Chloroplast-Archaea-Eukaryota-unknown)

phylotype()

make.biom()

count.groups()

deunique.seqs()

split.groups()

QIIME (v.1.7.0)

add_qiime_labels.py ()

pick_de_novo_otus.py ()

core_diversity_analyses.py (-e 750)

LEfSe (v.1.0)

"(Kruskal-Wallis test: alpha=0.05; Wilcoxon test: alpha=0.05; LDA threshold=2.0)"
