# Supplementary material for: Propionibacterium acnes overabundance and natural killer group 2 member D system activation in corpus‐dominant lymphocytic gastritis
Source: J Pathol. 2016 Oct 21;240(4):425–36. doi: 10.1002/path.4782 (PMC5111592; doi:10.1002/path.4782)
Supplement: Supplementary file 2 — Supplementary figure legends [file PATH-240-425-s002.docx]

**Supplemental figure legends**

**Figure S1.** **Differences at phylum level between healthy controls, HpG and LyG.** Relative abundance of *Actinobacteria* is significantly increased in LyG compared to healthy controls and HpG. Relative abundance of *Proteobacteria* is significantly increased in HpG compared to healthy controls and LyG samples. Relative abundance of *Bacteroidetes* is significantly decreased in HpG and LyG. Relative abundance of *Firmicutes* is significantly decreased in HpG. Data represent the mean ± SEM. **p<0.01, ***p<0.001, ****p<0.0001 by one-way ANOVA and *post-hoc* Bonferroni’s test.

**Figure S2. Validation of NGS sequencing results by qPCR.** Spearman correlation analysis (non-parametric) of samples with paired 16S rRNA gene sequencing and qPCR data shows a significant correlation of relative abundance and load (Ct value).

**Figure S3.** **NKG2DL expression in corpus biopsies** **measured by qRT-PCR.** *micb* mRNA is significantly increased in HpG samples compared to LyG. Among the ULBPs, *ulbp1* and *ulbp4* are down-regulated in LyG compared to control, while HpG samples show similar expression levels as healthy controls. (n=10). Data represent the mean ± SEM. *p<0.05, by One-way ANOVA and *post-hoc* Tukey’s test.

**Figure S4.** **AGS cell challenge for 4 h and bacterial viable cell counts.** **(A)** *mica*, *micb* and *il15* mRNA gene expression in AGS cells after 4h of infection with *P. acnes* and *E. coli* strains **(B)** Colony-forming-units per ml (CFU/ml) of P. acnes, H. pylori and E. coli (DSM 30083) strains after 24 h of co-cultivation with AGS (left) or MKN28 (right) cells. Bars show the mean ±SD, by One-way ANOVA and post-hoc Tukey’s test. ns: not significant

**Figure S5.** **Apoptosis and live/dead staining assay.** **(A)** AGS and **(B)** MKN28 cells were infected with different bacteria (*E. coli* denotes DSM30083) for 24h and assessed by Annexin V/PI staining and flow cytometry. **(C)** AGS and **(D)** MKN28 cells were stimulated with 5 mM of different SCFAs or HCl for 4h and assessed by Annexin V/PI staining and flow cytometry. Bar charts represent three independent Annexin V/PI experiments showing the percentage of viable (Annexin V-/PI-), apoptotic (Annexin V+/PI-) and dead (Annexin V+/PI+) cells, respectively. Bars show the mean ±SD. *p<0.05, ** p<0.001,***p<0.000, by one-way ANOVA and post-hoc Dunnett's test.
