# Supplementary figures and images for: Propionibacterium acnes overabundance and natural killer group 2 member D system activation in corpus‐dominant lymphocytic gastritis
Source: J Pathol. 2016 Oct 21;240(4):425–36. doi: 10.1002/path.4782 (PMC5111592; doi:10.1002/path.4782)

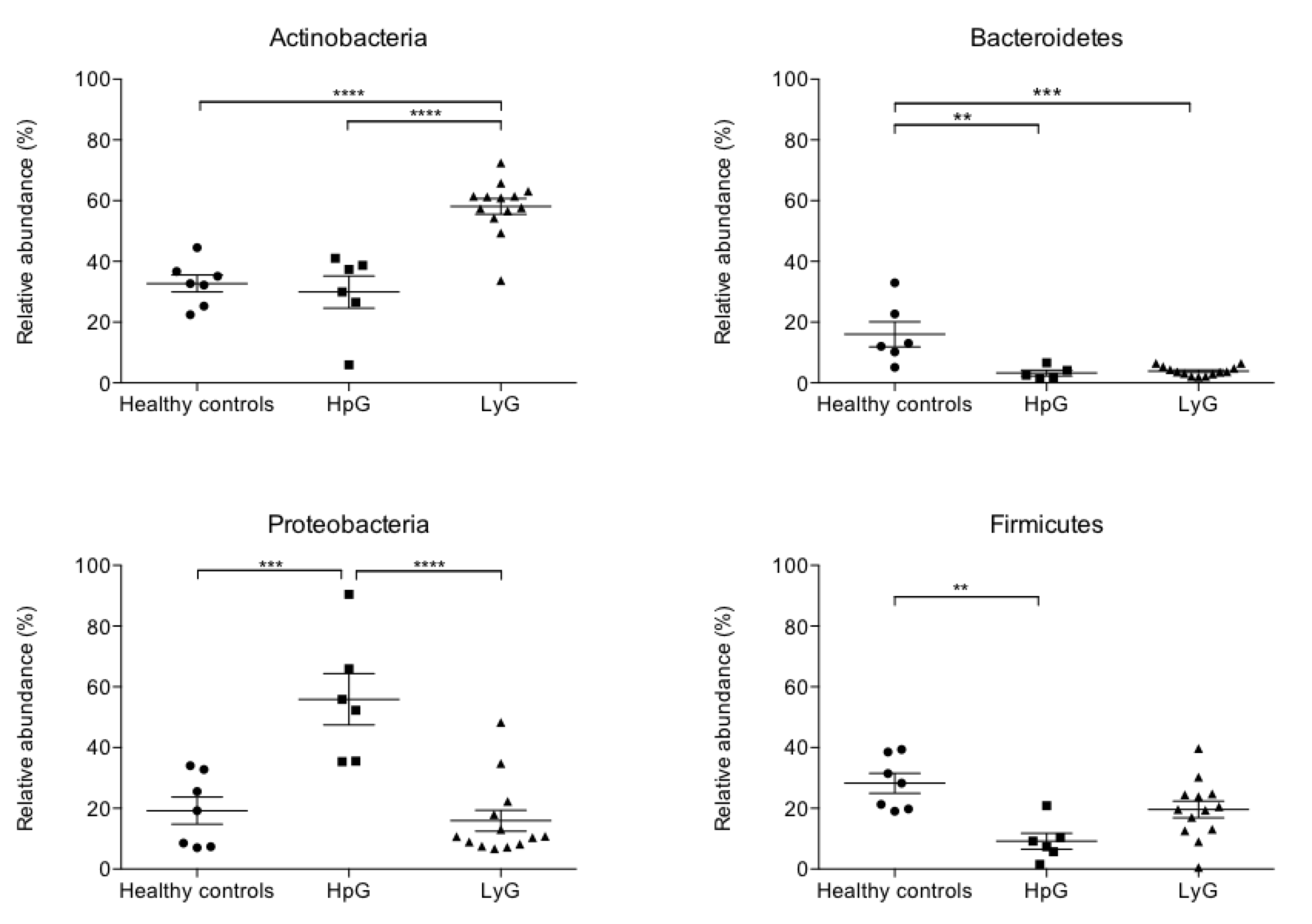

Supplement: Supplementary file 3 — Figure S1. Differences at phylum level between healthy controls, HpG and LyG. Relative abundance of Actinobacteria is significantly increased in LyG compared to healthy controls and HpG. Relative abundance of Proteobacteria is significantly increased in HpG compared to healthy controls and LyG samples. Relative abundance of Bacteroidetes is significantly decreased in HpG and LyG. Relative abundance of Firmicutes is significantly decreased in HpG. Data represent the mean ± SEM. **p<0.01, ***p<0.001, ****p<0.0001 by one‐way ANOVA and post‐hoc Bonferroni's test. [file PATH-240-425-s004.tiff]

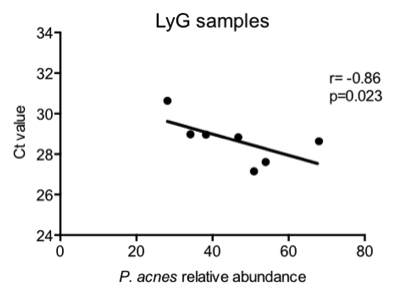

Supplement: Supplementary file 4 — Figure S2. Validation of NGS sequencing results by qPCR. Spearman correlation analysis (non‐parametric) of samples with paired 16S rRNA gene sequencing and qPCR data shows a significant correlation of relative abundance and load (Ct value). [file PATH-240-425-s007.tiff]

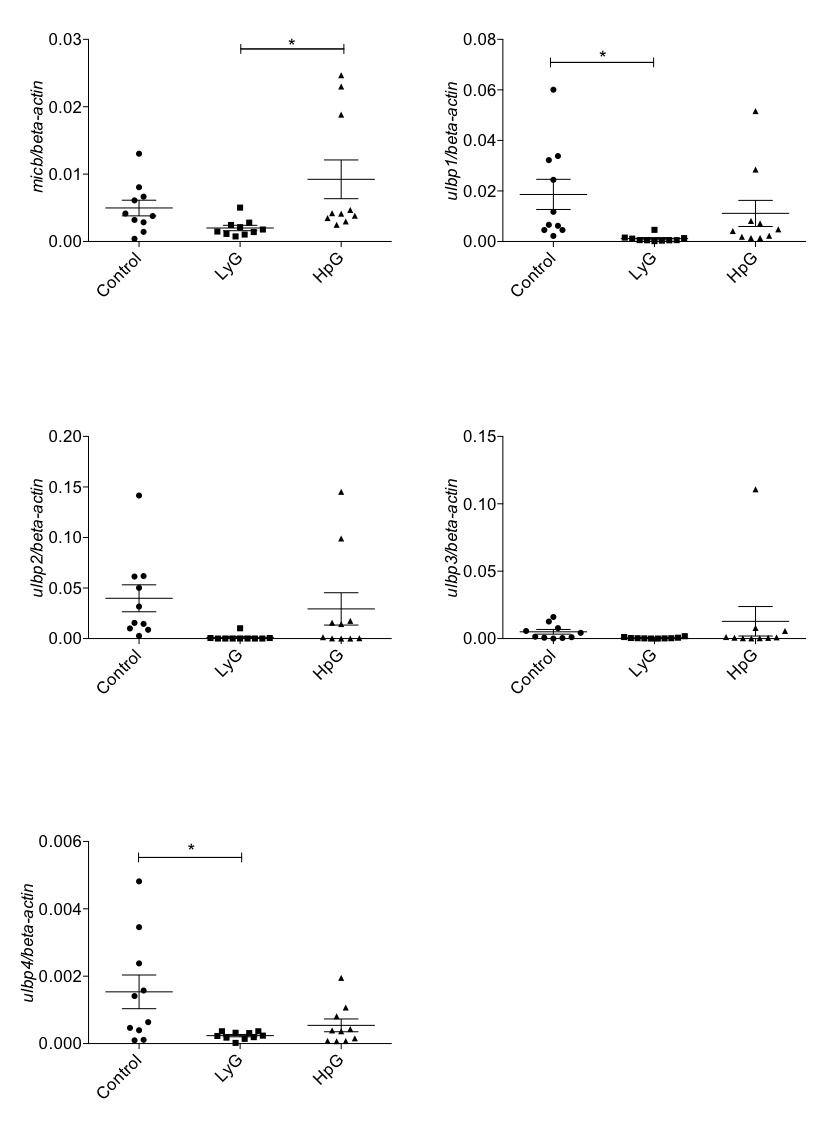

Supplement: Supplementary file 5 — Figure S3. NKG2DL expression in corpus biopsies measured by qRT‐PCR. micb mRNA is significantly increased in HpG samples compared to LyG. Among the ULBPs, ulbp1 and ulbp4 are down‐regulated in LyG compared to control, while HpG samples show similar expression levels as healthy controls. (n=10). Data represent the mean ± SEM. *p<0.05, by One‐way ANOVA and post‐hoc Tukey's test. [file PATH-240-425-s010.tiff]

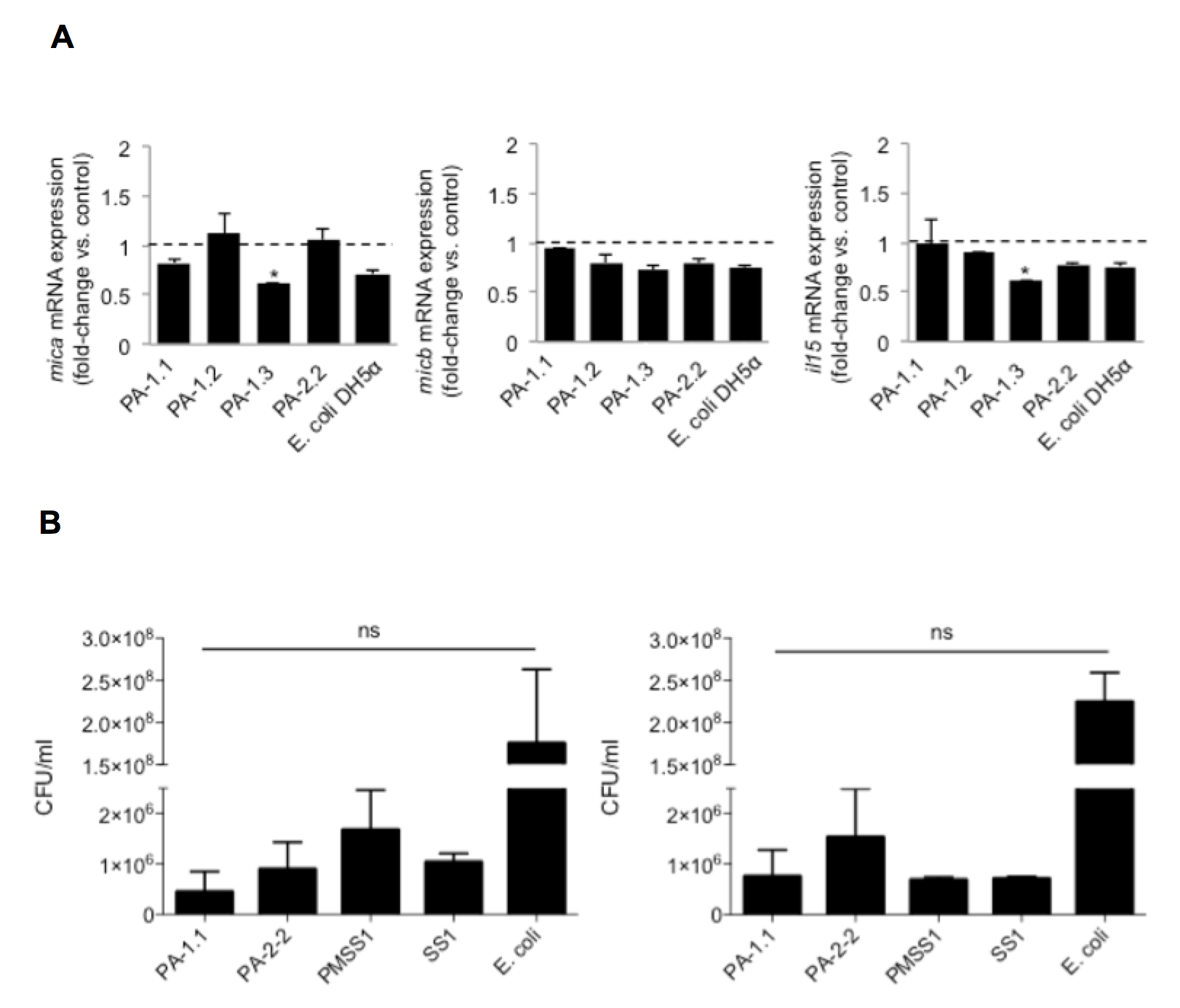

Supplement: Supplementary file 6 — Figure S4. AGS cell challenge for 4 h and bacterial viable cell counts. (A) mica, micb and il15 mRNA gene expression in AGS cells after 4h of infection with P. acnes and E. coli strains (B) Colony‐forming‐units per ml (CFU/ml) of P. acnes, H. pylori and E. coli (DSM 30083) strains after 24 h of co‐cultivation with AGS (left) or MKN28 (right) cells. Bars show the mean ±SD, by One‐way ANOVA and post‐hoc Tukey's test. ns: not significant [file PATH-240-425-s008.tiff]

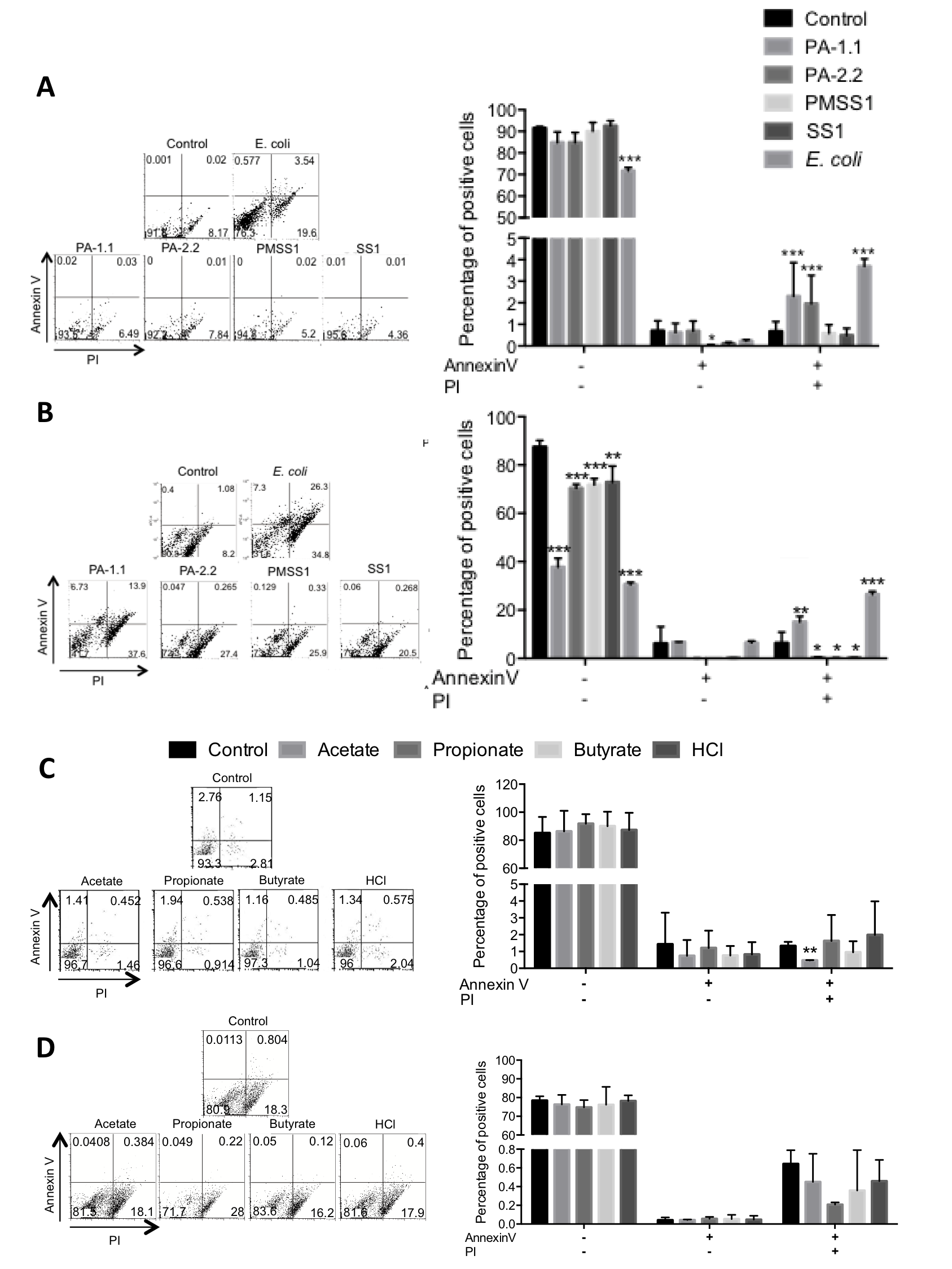

Supplement: Supplementary file 7 — Figure S5. Apoptosis and live/dead staining assay. (A) AGS and (B) MKN28 cells were infected with different bacteria (E. coli denotes DSM30083) for 24h and assessed by Annexin V/PI staining and flow cytometry. (C) AGS and (D) MKN28 cells were stimulated with 5 mM of different SCFAs or HCl for 4h and assessed by Annexin V/PI staining and flow cytometry. Bar charts represent three independent Annexin V/PI experiments showing the percentage of viable (Annexin V‐/PI‐), apoptotic (Annexin V+/PI‐) and dead (Annexin V+/PI+) cells, respectively. Bars show the mean ±SD. *p<0.05, ** p<0.001,***p<0.000, by one‐way ANOVA and post‐hoc Dunnett's test. [file PATH-240-425-s003.tiff]
